# Establishment of two serological methods for detecting IgG and neutralizing antibodies against Crimean-Congo hemorrhagic fever virus glycoprotein

**DOI:** 10.3389/fcimb.2024.1341332

**Published:** 2024-04-30

**Authors:** Qi Wang, Shen Wang, Zhikang Shi, Zhengrong Li, Yongkun Zhao, Na Feng, Tiecheng Wang, Feihu Yan, Xianzhu Xia

**Affiliations:** ^1^ College of Animal Science and Technology, Shihezi University, Shihezi, China; ^2^ Key Laboratory of Jilin Province for Zoonosis Prevention and Control, Changchun Veterinary Research Institute, Chinese Academy of Agricultural Sciences, Changchun, China; ^3^ College of Veterinary Medicine, Jilin University, Changchun, China

**Keywords:** CCHFV, rGP, rVSV/CCHFV, ELISA and sVNT, specific antibodies and serological samples

## Abstract

**Introduction:**

The Crimean-Congo hemorrhagic fever virus (CCHFV), the most geographically widespread tick-borne virus, is endemic in Africa, Eastern Europe and Asia, with infection resulting in mortality in up to 30% of cases. Currently, there are no approved vaccines or effective therapies available for CCHF. The CCHFV should only be manipulated in the BSL-4 laboratory, which has severely hampered basic seroprevalence studies.

**Methods:**

In the present study, two antibody detection methods in the forms of an enzyme-linked immunosorbent assay (ELISA) and a surrogate virus neutralization test (sPVNT) were developed using a recombinant glycoprotein (rGP) and a vesicular stomatitis virus (VSV)-based virus bearing the CCHFV recombinant glycoprotein (rVSV/CCHFV) in a biosafety level 2 (BSL-2) laboratory, respectively.

**Results:**

The rGP-based ELISA and rVSV/CCHFV-based sVNT were established by using the anti-CCHFV pre-G_C_ mAb 11E7, known as a broadly cross-reactive, potently neutralizing antibody, and their applications as diagnostic antigens were validated for the specific detection of CCHFV IgG and neutralizing antibodies in experimental animals. In two tests, mAb clone 11E7 (diluted at 1:163840 or 512) still displayed positive binding and neutralization, and the presence of antibodies (IgG and neutralizing) against the rGP and rVSV/CCHFV was also determined in the sera from the experimental animals. Both mAb 11E7 and animal sera showed a high reactivity to both antigens, indicating that bacterially expressed rGP and rVSV/CCHFV have good immunoreactivity. Apart from establishing two serological testing methods, their results also demonstrated an imperfect correlation between IgG and neutralizing antibodies.

**Discussion:**

Within this limited number of samples, the rGP and rVSV/CCHFV could be safe and convenient tools with significant potential for research on specific antibodies and serological samples.

## 1 Introduction

Crimean-Congo hemorrhagic fever (CCHF) is a tick-borne zoonosis that affects many countries in Africa, Europe, the Middle East, and Central Asia (Bente et al., 2013). As the causative agent of CCHF, Crimean-Congo hemorrhagic fever virus (CCHFV) belongs to the genus *Orthonairovirus*, family *Nairoviridae*, and order *Bunyavirales* (Garrison et al., 2020). Hard-bodied ticks belonging to the genus *Hyalomma* are the primary vectors of the CCHFV (Gargili et al., 2017). In addition to the major route of the CCHFV, which is the bite of infected ticks, contact with animals in the viremic phase and the blood of an infected patient in the acute phase of infection (Ergonul, 2012), vertical transmission, sexual contact, and laboratory-acquired accidental cases when handling viral material have been reported (Portillo et al., 2021). The CCHFV can infect humans and a wide range of wild or livestock animals (Capua, 1998). Clinical diseases like viral sepsis accompanied by bleeding from the gums, skin, nose, and gastrointestinal tract are restricted to humans, and their case fatality rates vary from 3% to 100% (Dzikwi-Emennaa et al., 2022). Moreover, there are also cases of transmission from wild and domestic animals to humans (Papa et al., 2013; Boushab et al., 2020). However, there is currently no approved vaccine, and treatment and control strategies include only avoiding direct or indirect contact with infected animals or humans. The potential seriousness of the CCHFV in people emphasizes the importance of human and veterinary surveillance and the need for safe serological antibody diagnoses. The traditional serological antibody diagnoses of CCHF can be achieved by enzyme-linked immunosorbent assay (ELISA) and the plaque reduction neutralization test (PRNT) to detect anti-CCHFV-specific antibodies. IgG ELISA is the most common technique for CCHFV antibody detection, which has been confirmed to have high sensitivity and specificity (Saijo et al., 2002). The virus neutralization assay is known as the “gold standard” sero-diagnostic assay to evaluate the antibody response to infection and vaccination of a wide variety of viruses associated with human diseases (Fukushi et al., 2012), and a neutralizing antibody is a reliable indicator of protective immunity against the CCHFV (Rodriguez et al., 2019). However, IgG ELISAs frequently use antigens derived from inactivated viruses that are propagated in the cells or brain tissues of suckling mice, and PRNT involves the manipulation of live CCHFV, which requires professional operators under biosafety level 4 (BSL-4) containment available in only a limited number of laboratories. Therefore, basic studies in serology on the CCHFV have been severely hampered by biosafety requirements. In 2019, the World Health Organization’s “WHO R&D Blueprint: Priority Diagnostics for CCHF” documentation prioritized the need for the development and validation of new serological tests (WHO, 2019). ELISAs based on recombinant proteins and neutralization assays using the pseudo virus may be advantageous in detecting serum samples and inhibitors in a biosafety level 2 (BSL-2) laboratory because they readily meet the need for a simple, inexpensive, reliable system for serological detections of viral hemorrhagic fevers (VHFs) (Bukbuk et al., 2014). Affordable, suitable alternatives to serological detections may be used for testing large numbers of serum samples, evaluating antibody response to vaccination, and even exploring the relation between IgG and the neutralizing antibody.

The CCHFV is a negative-sense single-stranded RNA virus, and its genome comprises three particular fragments, named small (S), medium (M), and large (L), that encode nucleocapsid protein (NP), glycoprotein (GP), and RNA-dependent RNA polymerase, respectively (Bergeron et al., 2010). The glycoprotein precursor (GPC) is cleaved and modified to generate the structural proteins G_N_ and Gc and three other domains: a variable mucin-like domain, a GP38 domain, and an NS_M_ domain (Bergeron et al., 2007). Structural glycoproteins (G_N_ and Gc) form spikes on the envelope and interact with host receptors to initiate infection. Glycoprotein is the primary target of the neutralizing antibodies (Bertolotti-Ciarlet et al., 2005) and is considered to be the immunodominant protein of the CCHFV. IgG antibody detection tests based on the glycoproteins of members of *Bunyaviridae* family, such as the Rift Valley fever (RVF) and Toscana virus, were conducted (Di Bonito et al., 2002; Jackel et al., 2013), and the Gn based ELISA against Rift Valley fever virus (RVFV) displayed a high and reliable specificity of more than 95% in the high number of tested field samples (Jackel et al., 2013). Therefore, glycoproteins are also important immunogens in ELISA methods, and there are few descriptions of the ELISA method based on the only glycoprotein of the CCHFV. High yields of protein are necessary for the preparation of serological reagents, and our initial attempts to express a recombinant CCHFV glycoprotein from a bacterial expression system using an entire GP gene were not successful. The truncated GP fragment (at aa 1443–1566 in the M segment of the IbAr10200 strain) has been identified as a highly conserved antibody-binding site in many virus strains and could bind to monoclonal antibody (mAb) 11E7 (Ahmed et al., 2005), which was a broadly cross-reactive, potently neutralizing antibody and protected suckling mice from a lethal CCHFV challenge (Bertolotti-Ciarlet et al., 2005). Thus, this region is very promising in having good reactogenicity and can be considered for detection.

As one simple and economical surrogate virus system, a reverse genetics system based on the vesicular stomatitis virus (VSV) has a high packaging efficiency and strong amplification ability (Fukushi et al., 2005), and types of recombinant VSVs (rVSVs) including replication-competent and replication-incompetent rVSVs can be used safely in BSL-2 laboratories (Liu et al., 2021). Moreover, a series of rVSVs expressing exogenous proteins have the properties of a receptor-mediated membrane fusion reaction similar to those of the enveloped GP of wild-type VSV. They have been constructed and replaced the traditional assays based on live viruses for the diagnosis and serological study of VHFs with high sensitivity (Haglund et al., 2000; Kohl et al., 2007; Bukbuk et al., 2014; Marzi et al., 2015; Rodriguez et al., 2019; Li et al., 2020; He et al., 2023). Therefore, developing a surrogate virus neutralization test (sVNT) using a VSV-based virus bearing the CCHFV recombinant glycoprotein is promising and needed.

Following these lines of thought, the aim of our study was to express and purify a truncated recombinant glycoprotein (rGP) and establish a recombinant VSV bearing the CCHFV recombinant glycoprotein. The truncated rGP and recombinant virus could be used as antigens to establish a novel indirect ELISA and an sVNT for serological detections, as well as to explore the relation between IgG and neutralizing antibodies. For this purpose, the immunoreactivity of two recombinant antigens was determined using mAb 11E7; meanwhile, two established tests were applied to study specific antibody responses induced by two CCHFV vaccine candidates currently undergoing research and development.

## 2 Materials and methods

### 2.1 Biosafety and ethics statement

The studies on the sVNT using the recombinant virus (rVSV/CCHFV) were performed under BSL-2 conditions. All rodent experiments were strictly conducted according to the guidance of the animal welfare and ethics committee of the Changchun Veterinary Research Institute, Chinese Academy of Agricultural Sciences. cDNA encoding the open reading frame of the CCHFV IbAr10200 strain GPC (GenBank ID: AF467768) was synthesized by Sangon Biotech (Shanghai) Co., Ltd. (Shanghai, China).

### 2.2 Cells, plasmid, antibodies, and serum samples

BSR-T7 cells (ATCC, CCL-10), VeroE6 cells (ATCC CRL-1586) were cultured in Dulbecco’s Modified Eagle’s Medium (DMEM, Gibco,Grand Island, NY, USA) supplemented with 10% fetal bovine serum (FBS, Gibco, Grand Island, NY, USA), 1% l-glutamine and 1% penicillin-streptomycin solution (P/S,Gibco, Grand Island, NY, USA) at 37°C with 5% CO_2._ The full-length plasmid (p3.1-VSV-eGFP) and supporting plasmids (p3.1-VSV-N, p3.1-VSV-P, p3.1-VSV-L, and p3.1-VSV-G) were prepared and stored in our laboratory, and all construction of plasmids were confirmed by sequencing in Comate Bioscience Co., Ltd. (Changchun, Jinlin, China). The mouse monoclonal antibody clone 11E7 anti-CCHFV pre-Gc was bought from BEI Resources (Manassas, VA, USA), and mouse serum anti-CCHFV-eGN was prepared and stored in our laboratory.

Fifty experimental serum samples (prepared and stored in our laboratory) were used in the present study. The experimental animal samples were derived from two vaccine candidates [G_C_ subunit vaccine (Wang et al., 2022a) and CCHFV candidate vaccine based on VSV (Rodriguez et al., 2019)], which have been reported. Ten experimental serum samples from BALB/c mice (at 6–8 weeks of age, five per group) were vaccinated with A-G-eG_C_ (5 μg) through subcutaneous (s.c.) injection two and three times. Twenty experimental serum samples from BALB/c mice (at 6–8 weeks of age, five per group) were vaccinated with CCHFV candidate vaccine based on VSV (0.5 × 10^6^ TCID_50_/mouse) through subcutaneous (i.s.) or intraperitoneal (i.p.) injection for one and two times. Ten experimental serum samples from Syrian hamsters (at 6–8 weeks of age, n = 5) were intraperitoneally (i.p.) immunized with CCHFV candidate vaccine based on VSV (2×10^6^ TCID_50_/Syrian hamster) one and two times. In addition, there were five experimental serum samples in the control groups [mice were inoculated with phosphate-buffered saline (PBS), 500 μL/mouse]. Five experimental serum samples in the control groups (Syrian hamsters) were inoculated with PBS (2 mL/Syrian hamster). Serum samples were incubated at 56°C for 30 min to inactivate potential viruses.

### 2.3 Establishment of recombinant glycoprotein-based ELISA

#### 2.3.1 Construction of plasmids, expression, and purification of truncated rGP

The constructions of plasmids and expression, purification, and identification of the rGP were conducted. During PCR, gene-specific primers as listed in **Table 1**, with restriction enzyme sites *Bam*HI and *Xho*I added upstream and downstream, respectively, were used for the amplification of the target gene, which was a 372-bp nucleotide segment between aa 1443 and 1566 (124aa) of the CCHFV IbAr10200 strain GPC (GenBank ID: AF467768) genome. The amplified gene fragment was connected by restriction enzyme sites to the linearized pET30a(+) vector with poly-histidine (His) tags, and the recombinant plasmid was sequenced and transformed into *Escherichia coli* BL21 Star (DE3) pLysS chemically competent cells. Finally, 500 mL *E. coli* containing the pET30a(+) vector-rGP plasmid was induced with 0.5 mM isopropyl-β-d-thiogalactoside (IPTG; Sigma, St. Louis, MO, USA), vigorously shaken overnight at 37°C for 6 h, and then harvested by centrifugation at 6,000 × *g* for 30 min. According to the manufacturer’s protocol of the HisPur Ni-NTA Spin Column (Thermo Fisher Scientific Inc., Waltham, MA, USA), recombinant protein was harvested and concentrated with a Vivaspin filter unit (Sartorius, Göttingen, Germany) at 4°C. The collected rGP was resuspended in 1× PBS buffer to reach a final concentration of 1 mg/mL and stored at −80°C.

**Table 1:** Oligonucleotide primers used in this study.

Oligonucleotide primer sequence (5′–3′)		Enzyme site
F^1 ^	CGC *GGATCC* TCAGGCTTAAAATTTGCAAGC	*Bam*HI
R^1^	CCG *CTCGAG* GCAAGTGCTGTTTTGCTTTCCG	*Xho*I

^1^Restriction enzyme sites are underlined and italicized.

#### 2.3.2 SDS-PAGE and Western blotting analyses

The molecular weight of the truncated rGP was determined using sodium dodecyl sulfate–polyacrylamide gel electrophoresis (SDS-PAGE) and Western blotting and described as follows. Two BeyoGel™ Plus PAGE 12% tris-acetate protein gels (Beyotime, Shanghai, China) were run at a constant 130 V for 90 min: one was stained with Coomassie brilliant blue (Beyotime, Shanghai, China), and the other was transferred onto the nitrocellulose membrane (Beyotime, Shanghai, China) according to the manufacturer’s instructions. After the immobilization and blocking of the bands, the membrane was incubated overnight at 4°C with the anti-CCHFV pre-G_C_ mAb clone 11E7 (BEI Resources, Manassas, VA, USA). After washing three times with PBST, the membrane was treated with horseradish peroxidase (HRP)-conjugated anti-mouse-specific IgG antibody (Bioworld, Minnesota, MN, USA; diluted 1:5,000) at room temperature for 1 h. After another three washes with PBST, the bands were visualized after incubation with DAB reagent (Thermo Fisher Scientific Inc., Waltham, MA, USA) according to the manufacturer’s instructions.

#### 2.3.3 Establishment of truncated rGP-based IgG ELISA

In this study, IgG ELISA using recombinant rGP was developed, and checkerboard titrations of rGP were used to optimize the ELISA protocol (Samudzi et al., 2012). Briefly, serial dilutions of antigen (2, 4, 8, and 10 μg/mL) were tested with ELISA using anti-CCHFV pre-G_C_ mAb 11E7 (BEI Resources, Manassas, VA, USA) to determine the concentration of rGP to be used as antigen. Then, MaxiSorp 96-well plates (Corning-Costar, Corning, NY, USA) were coated overnight at 4°C with 100 μL of purified rGP at a final concentration of 4 µg/mL. The next day, each well of the plates was washed three times with 200 μL PBS containing 0.05% Tween 20 (PBST) and then blocked with 150 μL of ddH_2_O containing 3% bovine serum albumin (BSA; Sigma, St. Louis, MO, USA) for 1 h at 37°C. The plates were washed three times with PBST and then incubated with the mAb clone 11E7 and control serum (mouse serum anti-PBS, prepared and stored in our laboratory) in duplicate, which were serially diluted twofold starting at a dilution of 1:80 with 1% BSA buffer. After a 1.5-h incubation period, the plates were washed three times with PBST and then were incubated with anti-mouse IgG-HRP antibody (1:20,000 dilution; Bioworld, St. Louis, MO, USA). After a further 1.5-h incubation period, the plates were washed, and 100 μL tetramethyl benzidine substrate (TMB; Sigma, St. Louis, MO, USA) was added to each well. The plates were incubated for 5–10 min at room temperature, and the reaction was stopped by adding 0.5 M H_2_SO_4_ (Sigma, St. Louis, MO, USA); the plates were read at 450 nm using an ELISA reader (Bio-Rad, Hercules, CA, USA). For data analyses, at the maximal ratio of P/N (OD_positive-mAb clone 11E7_/OD_negative-control_), the corresponding antigen concentration was identified as the optimum concentration of the rGP with the highest level of binding. The IgG response was considered to be positive if the mean absorbance of the mAb clone 11E7 was twofold greater than the mean absorbance of the same dilution of control serum, and titers were determined as the highest dilution at which the mean absorbance of the mAb clone 11E7 was twofold greater than the mean absorbance of the same dilution of control serum.

### 2.4 Development of a neutralization assay-based recombinant vesicular stomatitis virus bearing the CCHFV recombinant glycoprotein

#### 2.4.1 Construction of full-length cDNA clones and rescue of recombinant virus

**Figure 1:**
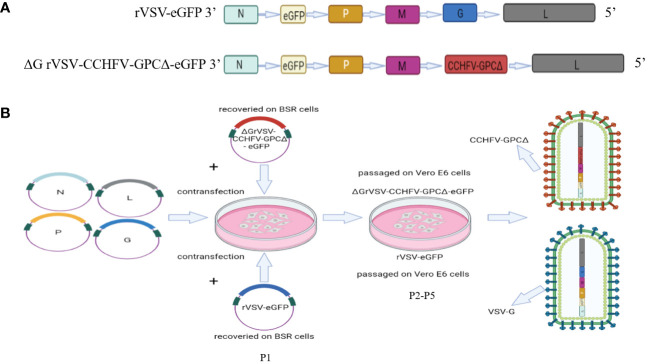
Schematic representation of the design and rescue of the rVSV-eGFP and ΔGrVSV-CCHFV-GPCΔ-eGFP (rVSV/CCHFV). **(A)** Genome organization comparing the VSV (wild-type) genome (rVSV-eGFP) and the rVSV vector expressing the CCHFV-GPCΔ open reading frame (rVSV/CCHFV). N: nucleoprotein, P: phosphoprotein, M: matrix protein, and L: large polymerase protein, eGFP; enhanced green fluorescent protein. The rVSV-eGFP had the VSV glycoprotein open reading frame (blue rectangle), which was exchanged with the open reading frame coding for the truncated glycoprotein precursor gene (GPCΔ, red rectangle) of CCHFV in the rVSV/CCHFV. **(B)** Generating a replication-competent virus: BSR-T7 cells were transfected with the p3.1-ΔGVSV-GPCΔ-eGFP plasmid and four supporting plasmids of VSV in-cluding p3.1-VSV-N, p3.1-VSV-P, p3.1-VSV-L and p3.1-VSV-G. The VSV-G*-rVSV/CCHFV particle was rescued with the VSV-G* (VSV-G*-rVSV/CCHFV (P1), and multiple passages (P2~P5) of the VSV-G*-rVSV/CCHFV on Vero E6 cells without VSV-G* resulted in a replication- competent rVSV/CCHFV (P5), in which the VSV-G gene was replaced by the GPCΔ gene of CCHFV. Finally, this replication-competent virus could effectively replicate on Vero E6 cells without VSV-G*. We used the P5 generation as the seed virus for the mass production of rVSV/CCHFV, and the P6 was used in the present study.

The reverse genetic operating system of the vesicular stomatitis virus Indiana strain (GenBank ID: nc_001560.1) has been described previously (Wang et al., 2022b). In the full-length plasmid p3.1-VSV-eGFP, a removable transcription unit encoding an enhanced green fluorescence protein (eGFP) (GenBank ID: hv192862.1) was added between the N and P genes of the VSV genome. In the present study, the plasmid p3.1-VSV-eGFP was used as the backbone, and cDNA encoding the open reading frame of the CCHFV IbAr10200 strain GPC (GenBank ID: AF467768) was synthesized and cloned into the p3.1-VSV-eGFP plasmid to generate the recombinant plasmid named p3.1-ΔGVSV-GPC-eGFP (rVSV/CCHFV). To increase the titer of the recombinant virus rVSV/CCHFV, the 53 amino acids of the C-terminal tail (aa 1632–1684) in CCHFV GPC were truncated. Finally, the coding sequence of VSV spike G was replaced by the CCHFV GPCΔ53aa (GPCΔ) in the recombinant plasmid-rVSV/CCHFV (**Figure 1A**).

The rVSV/CCHFV was recovered as described previously (Wang, Zhang et al., 2022). Briefly, BSR-T7 cells were transfected with 1.25 μg of the p3.1-ΔGVSV-GPCΔ-eGFP plasmid and four supporting plasmids of VSV comprising p3.1-VSV-N (0.75 μg), p3.1-VSV-P (1.25 μg), p3.1-VSV-L (0.25 μg), and p3.1-VSV-G (2μg). The procedure was performed according to the calcium phosphate transfection kit instructions (Invitrogen, Waltham, MA, USA), and the recovery of the virus was determined using cytopathic effects and eGFP reporter expression. A total of 48 h after transfection, the recombinant virus in the supernatant was harvested [first generation (P1)], which was passaged on Vero E6 cells from P2 to P5 (**Figure 1B**). The P5 generation of the virus supernatant was used as a seed virus and was stored at −80°C for future use.

#### 2.4.2 Identification of the replication-competent recombinant virus (rVSV/CCHFV)

##### 2.4.2.1 Electron microscopy analysis

To investigate whether the ultrastructure of rVSV-eGFP (“wild-type” VSV electron microscopy control) and rVSV/CCHFV displayed some differences, transmission electron microscopy studies were conducted. Freeze-thawed rVSV-eGFP and rVSV/CCHFV (P6) were centrifugated at 3,000 r/min for 10 min to remove the cell fragments. The collecting supernatants were negatively stained with uranyl acetate (Sigma, St. Louis, MO, USA) and then examined using an electron microscope.

##### 2.4.2.2 Growth kinetics of the rVSV/CCHFV

To assess the growth kinetics of the rVSVs (rVSV-eGFP or rVSV/CCHFV), Vero E6 cells with 80% confluence were incubated with rVSV/CCHFV (P6) at a multiplicity of infection (MOI) of 0.1, as well as rVSV-eGFP (as a positive control). After virus adsorption for 1 h at 37°C, the culture media were replaced with DMEM containing 5% FBS. The rVSV/CCHFV and rVSV-eGFP-containing supernatants were collected every 12 h after infection, serially diluted 10-fold in DMEM, and added together with Vero E6 cells into 96-well plates. The 96-well plates were incubated for 12–48 h at 37°C and observed under a fluorescence microscope (Zeiss, Oberkochen, Germany). Titrations of viruses in the supernatant infected with rVSVs from 12 to 96 h were calculated using the Reed and Muench method and expressed as TCID_50_/mL of stock.

##### 2.4.2.3 RT-PCR

The recombinant virus rVSV/CCHFV in the supernatant from P5 to P10 was harvested, and the expression of the CCHFV GPCΔ gene in the recombinant virus was identified by RT-PCR. The genome of the virus was extracted according to the manufacturer’s instructions (TIANGEN, Beijing, China). An RT-PCR was performed using TransGen^®^ II One-Step RT-PCR Super Mix Premix Ex Taq (TransGen Biotech, Beijing, China) with the following primers and probes: GF (5′-ATGCATATATCATTAATGTATGC-3′); GR (5′-CTAGCCTCTGGTTCTTCTACAACATT T-3′). The RT-PCR system and conditions are shown in **Table 2**.

**Table 2:** RT-PCR system.

Component	Volume
RNA template	8.8 μL
Forward primer (10 μM)	0.4 μL
Reverse primer (10 μM)	0.4 μL
2×TS One-Step Reaction Mix	10 μL
*TransScript* ^®^ One-Step Enzyme Mix	0.4 μL
Total volume	20 μL

Reaction conditions: 45°C 30 min; 94°C 5 min (94°C 30 s, 57°C 30 s, 72°C 5 min; 35 cycles); 72°C 10 min.

##### 2.4.2.4 Western blotting

In order to examine the expression of the CCHFV GPCΔ protein, Vero E6 cells infected with rVSV-eGFP (control) and rVSV/CCHFV for 72 h were centrifugated at 3,000 × *g* for 10 min, and the rVSVs (rVSV-eGFP and rVSV/CCHFV) in the supernatant were harvested. The collections were denatured in 5× loading buffer (Beyotime, Shanghai, China) and incubated at 95°C for 10 min before loading onto BeyoGel™ Plus PAGE 10% tris-acetate protein gels (Beyotime, Shanghai, China). According to the manufacturer’s instructions, the molecular weight of the GPCΔ protein was determined using Western blotting with two antibodies, which were a mouse serum anti-G_N_ (prepared and stored in our laboratory; diluted 1:200) and the anti-CCHFV pre-G_C_ mAb 11E7 (BEI Resources, Manassas, VA, USA, diluted 1:2,000).

##### 2.4.2.5 Immunofluorescence assay

To assess the expression of the CCHFV-GPCΔ gene in the rVSV/CCHFV, an immunofluorescence assay (IFA) was performed using specific antibodies. Briefly, Vero E6 cells were infected with rVSV/CCHFV and rVSV-eGFP (control) at a MOI of 0.1 and cultured in 24-well plates for 48 h at 37°C with 5% CO_2_. Then, the cells cultured in 24-well plates were fixed with 4% paraformaldehyde (Beyotime, Shanghai, China) at room temperature for 30 min and washed three times with PBS (containing 0.05% Tween 20, PBST). After being blocked with 3% bovine serum albumin (Sigma, St. Louis, MO, USA), cells were labeled with the primary antibody [a mouse serum anti-CCHFV-eG_N_, prepared and stored in our laboratory, 1:200 dilution; anti-CCHFV pre-G_C_ mAb 11E7 (BEI Resources, Manassas, VA, USA), 1:1,000 dilution] for 1 h at 37°C. After washing three times with PBST, cells were then incubated with the secondary antibody (Cy3-labeled anti-mouse IgG (H+L), Beyotime, Shanghai, China; 1:1,000 dilution) for 1 h at 37°C. After repeated washings, the cell nucleus was stained with 4′,6-diamidino-2-phenylindole (DAPI; Beyotime, Shanghai, China; 1:1,000 dilution) for 10 min. Finally, each well was washed three times with PBST and visualized using a fluorescence microscope.

#### 2.4.3 Establishment of neutralization assays based on rVSV/CCHFV

Handling of the CCHFV requires high-containment facilities of biosafety level 3 (BSL-3) and BSL-4 in endemic and non-endemic areas, respectively. In a certified BSL-2 laboratory, the rVSV/CCHFV particle was used to test the neutralizing antibody activity of anti-CCHFV pre-G_C_ mAb 11E7 (BEI Resources, Manassas, VA, USA), which was previously determined to have potently neutralizing activity (Zivcec, Metcalfe et al., 2015). First, twofold serial dilutions of anti-CCHFV pre-G_C_ mAb 11E7 and a mouse serum in the PBS group (initial dilution of 1:4, each dilution was performed in duplicate) were mixed with equal volumes of rVSV/CCHFV and rVSV/eGFP (a final concentration of 200 TCID_50_/mL), respectively, which were incubated at 37°C for 1 h as well as cell and viral controls. Second, the mixtures were loaded onto Vero E6 cells in a 96-well plate and incubated at 37°C for 48–72 h in a 5% CO_2_ environment. Finally, the fluorescence signal was observed using a fluorescence microscope, and 100% inhibition was considered positive in the sVNT.

### 2.5 Application of ELISA-based rGP and neutralization assay-based rVSV/CCHFV in experimental serum samples

Whether the ELISA-based rGP and neutralization assay-based ΔG rVSV-CCHFV-GPC-eGFP could be applied in the determination of IgG and neutralizing antibodies (NAbs) in animal sera was evaluated.

In the present study, 400 ng/well of the rGP and 200 TCID_50_ rVSV/CCHFV were used as the antigens in the IgG ELISA and sVNT, respectively. Twofold serial dilutions of animal sera (with 80-fold in the IgG ELISA and with fourfold in the initial dilution in the sVNT) were reacted with antigens in 96-well plates. Meanwhile, anti-CCHFV pre-G_C_ mAb clone 11E7 (BEI Resources, Manassas, VA, US) and sera in control groups in different dilutions were set as the positive and negative antibodies, respectively. Titration by endpoint dilutions was performed in serum samples. IgG response was considered to be positive if the mean absorbance of the sample was twofold greater than the mean absorbance of the same dilution of control serum. A total of 100% inhibition was considered positive in the sVNT. Data were analyzed as previously reported using GraphPad Prism 8.0. Data are shown as the mean ± SD and were analyzed using one-way ANOVA or the unpaired t-test (* p < 0.05, ** p < 0.01, *** p < 0.001, and **** p < 0.0001).

## 3 Results

### 3.1 Establishment of IgG ELISA assay based on truncated rGP

#### 3.1.1 Purification and identification of truncated rGP

**Figure 2:**
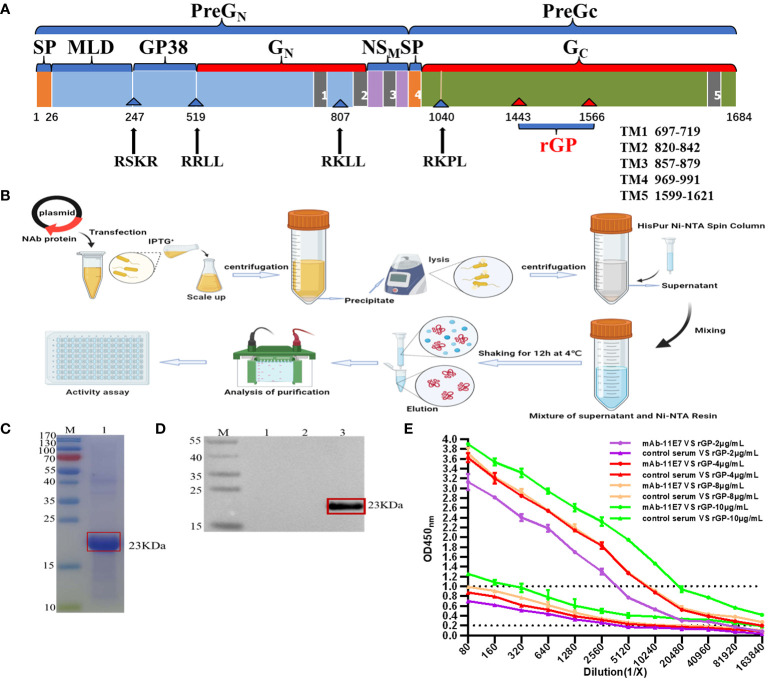
Expression of the truncated recombinant glycoprotein (rGP) and establishment of an IgG ELISA based on rGP. **(A)** A schematic representation of the glycoprotein open reading frame (ORF) of Crimean-Congo hemorrhagic fever virus (CCHFV) Ibar10200 strain. **(B)** Schematic diagram of expression, purification, and identification of recombinant protein in the prokaryotic expression system. **(C)** Sodium dodecyl sulfate–polyacrylamide gel electrophoresis (SDS-PAGE) identification of the rGP. The rGP was detected at 23 kDa boxed out in red. **(D)** Western blotting analysis of the rGP protein expression in *Escherichia coli*. Expression was detected with a mouse anti-CCHFV pre-GC monoclonal antibody (mAb) clone 11E7. (D-1) Uninduced IPTG-pET30a(+) empty carrier control. (D-2) Induced IPTG-pET30a(+) empty carrier control. (D-3) Target protein expressed in induced IPTG-pET30a(+) boxed out in red. **(E)** ELISA analysis of the binding ability of truncated GP (purified rGP, 2-4-8-10 μg/mL) to mAb clone 11E7.

**Figure 3:**
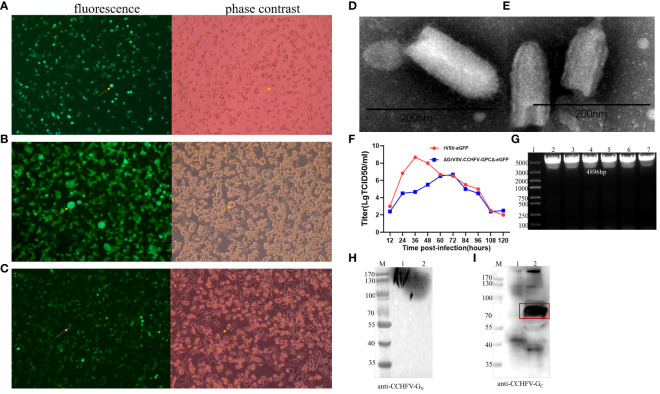
Identification analysis of recombinant vesicular stomatitis virus/Crimean-Congo hemorrhagic fever virus (rVSV/CCHFV). **(A)** The fluorescence and cytopathic effect (CPE) of rVSV-enhanced green fluorescence protein (eGFP) on Vero E6 cells. **(B)** The fluorescence and CPE of rVSV/CCHFV-P1 on BSR cells. **(C)** rVSV/CCHFV-P6 on Vero E6 cells at 48 h post-infection visualized under microscope. The fluorescence and CPE in panels **(A–C)** are indicated by a yellow arrowhead. **(D, E)** Electron microscopy (EM) detection of rVSVs. Transmission electron micrographs of rVSV-GFP **(D)** and replication-competent rVSV/CCHFV **(E)**. Supernatants were negatively stained with 2% aqueous uranyl acetate, and scale bars represent 200 nm. **(F)** Titer comparison of Vero E6 cells infected with rVSV-eGFP or rVSV/CCHFV at a multiplicity of infection (MOI) of 0.1 at hours 12, 24, 36, 48, 60, 72, 84, 96, 108, and 120. **(G)** Identification of CCHFV GPCΔ53aa gene in the rVSV/CCHFV (5th–10th generation) by RT-PCR. **(H, I)** Western blotting of approximately 30 μL rVSVs (10^6^ TCID_50_/mL) mixed with 5× loading buffer on 10% gradient TGX gels. 1, rVSV-eGFP; 2, rVSV/CCHFV. Particle preparations were incubated with a mouse serum anti-CCHFV-eG_N_ **(H)**, anti-CCHFV pre-G_r_ monoclonal antibody (mAb) clone 11E7 **(I)**, and a horseradish peroxidase (HRP)-conjugated secondary.

A schematic representation of the open reading frame (ORF) of CCHFV (Negeria- IbAr10200 strain) was clearly shown in **Figure 2A**. The result of the SDS-PAGE analysis showed that the truncated rGP was successfully expressed and purified through the HisPur Ni-NTA Spin Column (**Figure 2B**), presenting the apparent molecular weight of approximately 23 kDa (**Figure 2C**). The result of Western blotting analysis demonstrated that rGP could specifically react with the monoclonal antibody 11E7 (**Figure 2D**-3), and no expression of any immunoreactive protein was detected in mock preparations (**Figure 2D**-1, 2).

#### 3.1.2 Truncated GP-based IgG ELISA

A concentration of 4 μg/mL was selected for the detection work (**Figure 2E**), as the result of the maximal ratio of P/N (OD _positive-mAb clone 11E7_/OD _negative-control_). MAb clone 11E7 still showed a positive result (P/N > 2) when diluted in 163840 (**Figure 2D**), indicating that this truncated GP could be used as an antigen in the ELISA with a sensitive and specific binding activity.

### 3.2 Establishment of neutralization assays based on rVSV/CCHFV

#### 3.2.1 Generation and identification of CCHFV GPΔ53aa gene expression in the rVSV/CCHFV

Vero E6 cells infected with rVSV-eGFP were used as control (**Figure 3A**). After the recombinant plasmid-p3.1-ΔGVSV-GPCΔ-eGFP and four helper plasmids were co-transfected into BSR-T7 cells for 48 h, a replication-competent VSV-G*-rVSV/CCHFV (P1) was successfully rescued, with syncytial lesions and eventual cytopathic effect (CPE) in cell culture foci/plaque formations appearing on the monolayers, as well as green fluorescence under a fluorescent microscope (**Figure 3B**). Passaging of recombinant virus on Vero E6 cells (P2–P5) successfully resulted in a replication-competent virus rVSV/CCHFV. Vero E6 cells inoculated with rVSV/CCHFV (P6) supernatant presented green fluorescence under a fluorescent microscope and eventual CPE in cell culture foci/plaque formations on the monolayers (**Figure 3C**). These phenomena demonstrated that the replication-competent virus (rVSV/CCHFV) was successfully rescued, and the rVSV/CCHFV could effectively replicate on Vero E6 cells, which was consistent with the description that Vero E6 cells were susceptible to rVSV in a previous study (He et al., 2023).

In the electron microscopy analysis, both rVSV-eGFP (**Figure 3D**) and rVSV/CCHFV particles (**Figure 3E**) were observed between 170 and 200 nm and maintained rhabdovirus morphology, a classical bullet shape with coiled intra-virion structure. At a MOI = 0.1, the growth kinetics of the rVSV/CCHFV and rVSV-eGFP showed that rVSV-eGFP (wild-type control) peaked in the titer of 10^8.667^ TCID_50_/mL at approximately 36 hpi, while the peak titer of rVSV/CCHFV was 10^6.667^ TCID_50_/mL at 72 hpi (**Figure 3F**). The growth titer of rVSV/CCHFV was lower than that of rVSV-GFP due to the recombination of a larger foreign gene (**Figure 3F**). Viral genomes were extracted from the 5th to 10th generation of rVSV/CCHFV and assessed by RT-PCR. The result showed that there was a specific band at 4,896 bp in every generation, suggesting the expression of CCHFV GPCΔ (**Figure 3G**).

Western blotting analysis was performed to determine the status of CCHFV GPCΔ53aa expression using antibodies against CCHFV-G_N_ or Gc. The IbAr10200 strain of CCHFV structural glycoproteins G_N_ and G_C_ present as bands of approximately 37 kDa and 75 kDa, and the precursor molecule CCHFV-PreG_N_ and PreG_C_ present as bands of approximately 140 kDa and 85 kDa, respectively (Sanchez et al., 2002; Vincent et al., 2003; Sanchez et al., 2006). In the present study, the Western blotting data demonstrated that non-specific binding proteins were observed in rVSV-eGFP (lane 1, **Figure 3I**), and there was a strong band at 70–100 kDa recognized by the specific CCHFV-G_C_ antibody (anti-CCHFV pre-G_C_ mAb 11E7, BEI Resources, Manassas, VA, USA) in rVSV/CCHFV (lane 2, **Figure 3I**). This indicated that the specific expression of CCHFV GP was incorporated in rVSV/CCHFV virions presenting in the forms of the mature CCHFV-G_C_ and PreG_C_. However, the Western blotting using the antibody against CCHFV-G_N_ (a mouse serum anti-CCHFV-eG_N_, prepared and stored in our laboratory) showed that there were no specifically evident bands at approximately 37 kDa for the mature G_N_ or 140 kDa for the PreG_N_ (lane 2, **Figure 3H**). The phenomenon may be related to their expression concentrations, as only unconcentrated and purified rVSV/CCHFV was currently available. Our study demonstrated that the rVSV/CCHFV could express CCHFV-GPΔ *in vitro* and strongly express CCHFV-G_C_ or PreG_C_ in the virion, which was consistent with the description in a previous study (Rodriguez et al., 2019).

**Figure 4:**
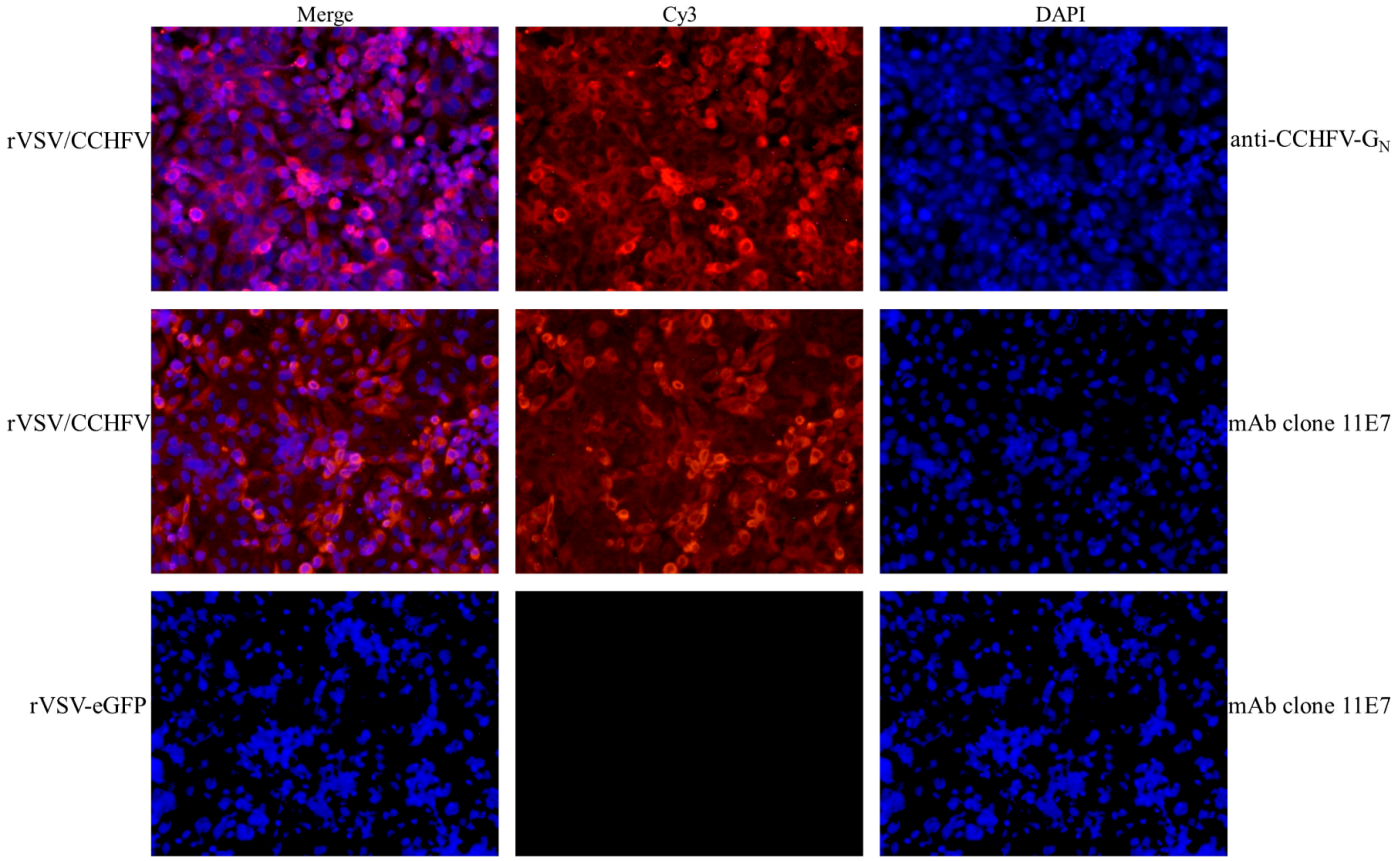
Indirect immunofluorescence staining to detect the expression of Crimean-Congo hemorrhagic fever virus (CCHFV) GP (100×). After being infected with recombinant vesicular stomatitis virus (rVSV) for 48 h, Vero E6 cells were permeabilized and immunostained with specific antibodies (anti-CCHFV-eG_N_ and anti-CCHFV pre-G_C_ monoclonal antibody (mAb) clone 11E7) and the control antibody [serum in phosphate-buffered saline (PBS) group]. Then, Vero E6 cells were stained with the anti-mouse Cy3-conjugated secondary antibody and 4′,6-diamidino-2-phenylindole (DAPI). Merge: DAPI + Cy3. DAPI, 4′,6-diamidino-2-phenylindole; Cy3, cyanine. Cytomembranes were stained with Cy3 (red for cytomembrane localization). Nuclei were stained with DAPI (blue for nuclear localization).

To assess the expression of the CCHFV-GPCΔ in the vector, an immunofluorescence assay was performed with antibodies against the CCHFV-G_C_ or CCHFV-G_N_. The result showed that rVSV/CCHFV was recognized by two CCHFV GP-specific antibodies (**Figure 4**, Cy3-rVSV/CCHFV), while rVSV-eGFP did not react with two CCHFV GP-specific antibodies (the reaction with mAb clone 11E7 was only shown here, **Figure 4**, Cy3-rVSV-eGFP). The assay revealed strong *in vitro* expression of both proteins. Moreover, the cell cytomembrane stained with Cy3 appeared red (**Figure 4**, Cy3-rVSV/CCHFV), the cell nuclei stained with DAPI appeared blue (**Figure 4**, DAPI-rVSV/CCHFV), and the surface of rVSV/CCHFV-infected cells appeared red (**Figure 4**, Merge-rVSV/CCHFV), suggesting that both proteins have efficiently packaged on the surface of rVSV/CCHFV.

In summary, the replication-competent virus (rVSV/CCHFV) was successfully rescued, and CCHFV-GPCΔ was strongly expressed *in vitro*.

#### 3.2.2 Establishment of rVSV/CCHFV-based neutralization assay

**Figure 5:**
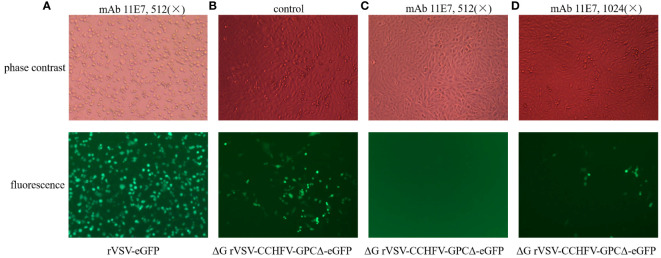
Establishment of recombinant vesicular stomatitis virus/Crimean-Congo hemorrhagic fever virus (rVSV/CCHFV)-based neutralization assay by evaluating the neutralization infectivity of monoclonal antibody (mAb) 11E7. **(A)** MAb 11E7 was unable to block rVSV-enhanced green fluorescence protein (eGFP) infection in Vero E6 cells [fluorescence, bottom; cytopathic effect (CPE), top]. **(B)** Sera in control group [serum in phosphate-buffered saline (PBS) group] were not able to inhibit the infection of rVSV/CCHFV particles in Vero E6 cells (fluorescence, bottom; CPE, top). **(C)** MAb 11E7 (at a dilution of 1:512) was able to completely inhibit the infectivity of rVSV/CCHFV particles (fluorescence, bottom; CPE, top). **(D)** MAb 11E7 (at a dilution of 1:1,024) cannot completely inhibit the infectivity of rVSV/CCHFV particles in Vero E6 cells (fluorescence, bottom; CPE, top); magnification of microscopy images, 100×.

The functionality of the rVSV/CCHFV particle was also evaluated using anti-CCHFV pre-G_C_ mAb 11E7 in a neutralization assay (BEI Resources, Manassas, VA, USA). No inhibition was observed after the reaction between rVSV-eGFP and the anti-CCHFV pre-G_C_ mAb clone 11E7 (**Figure 5A**), and sera in the control group (mice were vaccinated with PBS) were not able to inhibit rVSV/CCHFV particles in Vero E6 cells (**Figure 5B**), indicating the specificity of the responses. Inhibition of 100% was observed when rVSV/CCHFV particles were treated with anti-CCHFV pre-G_C_ mAb 11E7 (at a dilution of 1:512, BEI Resources, Manassas, VA, USA; **Figure 5C**), while the mAb 11E7 (at a dilution of 1,024) did not completely inhibit the infection of rVSV/CCHFV particle in Vero E6 cells (**Figure 5D**). The results indicated that the rVSV/CCHFV-based neutralization assay was effective in the neutralization test.

### 3.3 Application of the rGP-based ELISA and rVSV/CCHFV-based neutralization assay in experimental serum samples

rGP-based ELISA and the rVSV/CCHFV-based neutralization assays were used to determine the titers of anti-CCHFV-GP IgG and neutralizing antibodies in experimental serum samples. Meanwhile, the antigenicity (binding and neutralization capacity) of the rGP and rVSV/CCHFV particles was validated again by these experimental serum samples.

**Figure 6:**
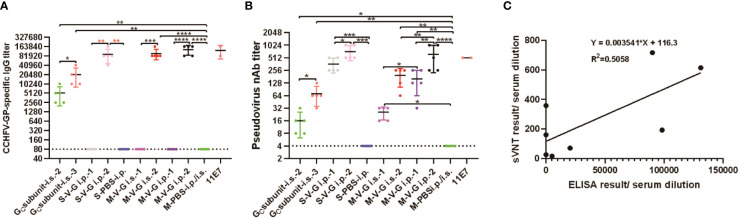
*In vivo* experiment humoral immune responses and correlation between surrogate virus neutralization test (sVNT) and indirect IgG ELISA. Sera from mice or Syrian hamsters vaccinated with G_C_ subunit candidate vaccine and Crimean-Congo hemorrhagic fever virus (CCHFV) candidate vaccine based on vesicular stomatitis virus (VSV). **(A)** Enzyme-linked immunosorbent assay (ELISA) using plates coated with 400 ng/well of purified recombinant glycoprotein (rGP) and diluted sera from mice or Syrian hamsters vaccinated with G_C_ subunit candidate vaccine and CCHFV candidate vaccine based on VSV. **(B)** Surrogate virus neutralization test (sVNT) from diluted endpoint mouse or Syrian hamster sera with G_C_ subunit candidate vaccine and CCHFV candidate vaccine based on VSV incubated on Vero E6 cells. Anti-CCHFV pre-GC monoclonal antibody (mAb) clone 11E7 (11E7) was used as a positive control and diluted in duplicate, together with mouse and Syrian hamster sera serially diluted twofold with DMEM. Data are shown as the mean ± SD and were analyzed using an unpaired t-test (* p < 0.05, ** p < 0.01, *** p < 0.001, and **** p < 0.0001). G_Csubunit i.s.-2/i.s.-3_: mice were vaccinated with subunit vaccine by subcutaneous injection after the second and third vaccinations. M-V-G_i.s.-1_ and M-V-G_i.s.-2_, M-V-G_i.P.-1_ and M-V-G_i.P.-2_: Mice were vaccinated with CCHFV candidate vaccine based on VSV by subcutaneous or intraperitoneal injection after the first and second vaccinations. S-V-G_i.p.-1_ and S-V-G_i.p.-2_: Syrian hamsters were vaccinated with CCHFV candidate vaccine based on VSV by intraperitoneal injection after the first and second vaccinations. S-PBS/M-PBS: mice or Syrian hamsters were vaccinated with phosphate-buffered saline (PBS). **(C)** ELISA results with serum dilutions (x-axis) and the sVNT result/serum dilution (y-axis). There is an imperfect correlation (R^2 ^= 0.5058) between the titer of indirect IgG ELISA and neutralizing antibodies.

As expected, the IgG antibody titers of five experimental serum samples in the control group (M-PBS) were below the limit of detection (**Figure 6A**). In the detection of mouse sera vaccinated with subunit vaccine, the second immunization of mice with the A-G-eG_C_ vaccine (G_C subunit i.s.-2_) through i.s. route produced significantly detectable IgG and NAbs than that in the M-PBS group, and the third immunization of mice with the A-G-eG_C_ vaccine (G_C subunit i.s.-3_) through i.s. route produced significantly detectable IgG and NAbs than that in the G_C subunit i.s.-2_ group (**Figures 6A, B**). In the testing of animal sera vaccinated with the CCHFV candidate vaccine based on VSV, the first immunization of mice with the rVSV/CCHFV vaccine by either i.s. or i.p. pathway (subcutaneous or intraperitoneal injection, M-V-G_i.s.-1_ or M-V-G_i.p.-1_, respectively) did not generate any detectable IgG antibodies (**Figure 6A**). Interestingly, these negative samples (M-V-G_i.s.-1_ or M-V-G_i.p.-1_) in the ELISA results were demonstrated as truly positive in the detection of NAbs (**Figure 6B**), with the geometric mean titer (GMT) reaching 25.6 ± SD (M-V-G_i.s.-1_), and the GMT was 160 ± SD in M-V-G_i.p.-1_. IgG antibody titers after the booster vaccination showed relatively high levels and were significantly higher than that after the first vaccination with two inoculation methods (*** p < 0.001), with GMT reaching 98,304 ± SD in the M-V-G_i.s.-2_ group and reaching 131,072 ± SD in the M-V-G_i.P.-2_ group (**Figure 6A**). In addition, NAbs after the booster vaccination (GMT was 614.4 ± SD) showed relatively high levels and were significantly higher than that after the first vaccination (160 ± SD) by intraperitoneal injection (** p < 0.01). Compared with the GMT of NAbs (192 ± SD) in the M-V-G_i.s.-2_ group, the GMT of NAbs in the M-V-G_i.P.-2_ group was significantly enhanced, reaching 614.4 ± SD (**Figure 6B**). Furthermore, the results also showed that the IgG antibody titers of the five experimental serum samples in the control group (S-PBS) were below the limit of detection (**Figure 6A**). There were no detected IgG antibodies in five experimental serum samples from the first immunization of Syrian hamsters with the rVSV/CCHFV vaccine by i.p. pathway (intraperitoneal injection, S-V-G_i.p.-1_; **Figure 6A**), while the GMTs of NAbs reached 358.4 ± SD, which were significantly higher than those in the control group (S-PBS, unpaired t-test, *** p < 0.001; **Figure 6B**). Compared with S-V-G_i.p.-1_, IgG antibody titers and NAbs of sera in S-V-G_i.p.-2_ were significantly enhanced, with the endpoint GMT of IgG titers and NAbs reaching 89,512 ± SD (unpaired t-test, **p < 0.01) and 716.8 ± SD (unpaired t-test, * p < 0.05) (**Figures 6A, B**). As the immunizing doses of mice and Syrian hamsters were different, no comparison was made about the serum antibody levels between the two animals.

Moreover, the overall results of the two serological assays remained consistent in that the highest neutralization efficiency was observed in the mouse serum sample with the highest IgG titer, which was also mentioned in a previous study (Vasmehjani et al., 2020). However, comparing the results of rGP-based ELISA and sVNT, we also found that some sera were positive in sVNT, whereas these sera were negative in the rGP-based ELISA. Because of the lack of testing with field samples, we could not make a perfect correlation (R^2^ = 0.5058) between the titer of CCHF IgG and neutralizing antibodies (**Figure 6C**), and this phenomenon demonstrated an inconsistent correlation between IgG and neutralizing antibodies. All controversial serum samples should be additionally tested with traditional detection methods, such as inactivated virus-based ELISA and live virus-based PRNT in the future.

Therefore, rGP and rVSV/CCHFV can be used to detect antigens to determine the titers of anti-CCHFV-GP IgG and neutralizing antibodies in experimental serum samples from two kinds of animals (mice and Syrian hamsters).

## 4 Discussion

Various serological assays including the detection of antibodies by ELISA (IgG and IgM), indirect IFA, hemagglutination inhibition test, and the virus neutralization test (VNT) can be used for the detection of CCHFV antibodies in human and animal sera. However, live CCHFV must be manipulated in BSL-4 laboratories, and basic seroprevalence studies on the CCHFV have been severely hampered by biosafety requirements. Seroprevalence studies, such as ELISAs based on recombinant proteins and VNTs based on surrogate viruses, are safe and comparably inexpensive detecting tools. NP is highly conserved and the immunodominant protein of CCHFV. However, even though NP is highly immunogenic, antibodies against the NP do not exhibit neutralizing activities. Currently, ELISAs based on CCHFV NP have been used for detecting IgG antibodies and not neutralizing antibodies in humans and animals (Saijo et al., 2002; Tang et al., 2003; Saijo et al., 2005). GP is a dominant protein that causes IgG and neutralizing antibodies, and more and more researchers have realized the role of GP in the detection of antibodies. Recently, some ELISAs based on GP and NP have been published, such as a multiplex assay for simultaneous detection of antibodies against CCHFV NP and glycoproteins (G_N_ ectodomain and GP38) in ruminants (Hoste et al., 2023), an ELISA (Rec-ELISA) based on a purified recombinant nucleocapsid protein (rNP) and mucin-like variable domain (rMLD) for detection of antibodies in CCHF-suspected patient sera (Gulce-Iz et al., 2021), and a Gc and NP-specific ELISA for detection of antibodies in domestic animal sera (Belij-Rammerstorfer et al., 2022). However, this sophisticated approach is hardly feasible in a normal lab. Moreover, many vaccine candidates based on glycoprotein have demonstrated variable efficacy in multiple animal models for CCHFV, while there are few descriptions of seroprevalence studies based on only CCHFV GP to evaluate the antibody response after vaccination and even explore the relation between IgG and the neutralizing antibody in the BSL-2 laboratory.

In our present study, we bacterially expressed a truncated glycoprotein to achieve a high yield, whose gene was a highly conserved antibody-binding site in the CCHFV GP. Purified rGP was demonstrated as a 23-kDa protein with antigenicity. Our data support that this region (aa 1443–1566 in the M segment of the IbAr10200 strain) is an antibody-binding site to mAb 11E7 and has good reactogenicity (Ahmed et al., 2005). Our bacterially expressed rGP is more safe and comparably inexpensive compared with traditional inactivated viruses and enables further production on a large scale. In addition, a recombinant virus based on the reverse genetic system of VSV was constructed, with the coding sequence of VSV spike G replaced by the CCHFV GPC and the 53 amino acids of C-terminal tail (aa 1632–1684) in CCHFV GPC truncated in the present study. The replication-competent virus (rVSV/CCHFV) was successfully rescued (the peak titer was 10^6.667^ TCID_50_/mL), and rVSV/CCHFV particle maintained the rhabdovirus morphology and a classical bullet shape. Our data support that the truncation of this region enables the replication of competent rVSV/CCHFV formation (Suda et al., 2016). CCHFV-GPCΔ was strongly expressed *in vitro*, which we were able to detect by Western blotting and IFA, and a form of CCHFV-G_C_ or PreG_C_ was present and functional on the surface of the rVSV/CCHFV virion with the tools currently available. Structural CCHFV-G_C_ has been known as an important protein for CCHFV and contains a putative receptor binding region for mammalian cell surface nucleolin (Xiao et al., 2011), the main neutralization epitope (Ahmed et al., 2005). As the only un-purified rVSV/CCHFV currently was available, the detecting of of the mature CCHFV-G_N_ or PreG_N_ was limited. Our replication-competent rVSV can propagate and display high infectivity on Vero E6 cells, enabling further explorations about these components at a BSL-2 containment without the need for transfections for in trans expression of CCHFV-GPC onto VSVΔG systems.

Two methods for the detection of antibodies in the forms of an ELISA and an sVNT were developed using a rGP and a recombinant virus rVSV/CCHFV, respectively. The binding and neutralization activity of the rGP and rVSV/CCHFV were also evaluated using anti-CCHFV pre-G_C_ mAb 11E7 (BEI Resources, Manassas, VA, USA), which was identified as a broadly neutralizing anti-CCHFV mAb in the study by Marko Zivcec et al (Zivcec et al., 2015). In two tests, 400 ng/well of the rGP and 200 TCID_50_ rVSV/CCHFV were used as the antigens in the IgG ELISA and sVNT. The results showed that mAb clone 11E7 (diluted at 1:163,840 or 512) still displayed positive binding and neutralization, indicating that bacterially expressed rGP and rVSV/CCHFV have good reactogenicity. The use of the broadly cross-reactive and neutralizing antibody mAb clone 11E7 (BEI Resources, Manassas, VA, USA) further strengthens the reliability and specificity of the assays.

As real CCHFV-neutralization assays involve the manipulation of live CCHFV and require professional operators under BSL-4 containment, which are not currently available in our laboratory, CCHFVpv (a pseudotyped VSV bearing the CCHFV envelope GP) showed similar neutralizing activities with the real CCHFV in the detection of six serum samples obtained from patients in Tajikistan with confirmed CCHF, and Chamberlain et al. thought that neutralization assays using CCHFVpv in BSL-2 laboratories can be used as a convenient alternative to assays using infectious CCHFV in BSL-4 facilities (Suda et al., 2018). In addition, the reliability of using recombinant VSV instead of the authentic virus to detect SARS-CoV-2 specific neutralizing antibodies has been shown in other studies (Case et al., 2020; Dieterle et al., 2020; Li et al., 2020; He et al., 2023). In the present study, the applications of rGP and rVSV/CCHFV as diagnostic antigens were validated for the specific detection of CCHFV IgG and neutralizing antibodies in experimental animals, indicating that the bacterially expressed rGP and rVSV/CCHFV have good immunoreactivity with animal sera. By analyzing and comparing the detection results of two vaccine candidates between this study and methods mentioned in the literature of Jeroen Kortekaas et al. and Sergio E. Rodriguez et al (Kortekaas et al., 2015). (Wang et al., 2022a), we found some similarity in the test results in the evaluation of the CCHFV candidate vaccine based on VSV, which can introduce high-titer antibody in mice, especially the neutralizing antibody. In addition, the immunizing dose did influence the neutralizing antibody produced by this VSV vector candidate vaccine. In the study of Sergio E. Rodriguez et al., they intraperitoneally administered 10^7^ pfu/dose of ΔG rVSV-CCHFV-GPCΔ to prime and boosted groups of five STAT-1^−/−^ mice; the result showed that the prime-only group had neutralizing antibody titers with a PRNT50 of <1:1,280 and that the boosted group had a PRNT50 of <1:320 at the study endpoint. In our study, we intraperitoneally administered 0.5 × 10^6^ TCD_50_/dose of (ΔG rVSV-CCHFV-GPCΔ) to prime and boosted groups of five BABL/c mice; NAbs after the booster vaccination (GMT was 614.4 ± SD) showed relatively high levels and were significantly higher than those after the first vaccination (160 ± SD) by intraperitoneal injection. Moreover, we found that the titer of antibodies produced by VSV-CCHFV-GP via the intraperitoneal route was significantly increased than that via the subcutaneous route, with the GMT of NAbs (192 ± SD) in the M-V-G_i.s.-2_ group and the GMT of NAbs (614.4 ± SD) in the M-V-G_i.P.-2_ group. The immunization route may be a possible explanation for the difference in neutralizing antibody titers between the previous G_C_ protein subunit vaccine (Jeroen Kortekaas et al.) via the intraperitoneal route (an average 80% endpoint titer of 333) and our GEM-PA-based subunit vaccine (G-eG_C_) of CCHFV via the subcutaneous route (an average neutralizing antibody titer of 1:70.4; Wang et al., 2022a). Currently, Syrian hamsters are a good animal model for evaluating the immunogenicity of the SARS-CoV-2 candidate vaccine based on VSV, and sVNT based on recombinant VSV is usually used for detecting the titers of neutralizing antibodies in Syrian hamster sera (Wang et al., 2022b; He et al., 2023). However, there have been no reports of this CCHFV candidate vaccine based on VSV being administered to Syrian hamsters, and we used the sVNT to detect specific antibodies in Syrian hamster sera vaccinated with CCHFV candidate vaccine based on VSV for the first time.

Two serological assays consistently had the highest neutralization efficiency observed in the serum sample with the highest IgG titer, which was also mentioned in a previous study (Vasmehjani et al., 2020). However, comparing the IgG antibody titers and NAbs in the activated animal sera involved in this study, we found that some sera after the first vaccination were positive in sVNT, whereas these sera were negative in the rGP-based ELISA, indicating an inconsistent correlation between IgG and neutralizing antibodies. Because of the lack of testing with field samples, we could not make a perfect correlation (R^2 ^= 0.5058) between the titer of CCHF IgG and neutralizing antibodies. Using the truncated recombinant GPC antigen does have its limitations, which may be the explanation for the different outcomes of the titers of specific IgG antibodies compared to the pseudo-neutralization assay. All controversial serum samples should be additionally tested in the future using traditional detection methods, such as inactivated virus-based ELISA and live virus-based PRNT. In addition, the relationship between IgG and neutralizing antibodies should be investigated to research the immune response dynamics and contribute to a better understanding of CCHFV infection in the future. Moreover, infected animals would have positive results in both proteins (NP and GP), whereas those immunized with a vaccine based on GP animals would have positive GP ELISAs and pseudo-neutralization assay. Therefore, the two assays represent a useful tool for vaccination strategies following the differentiating infected from vaccinated animals (DIVA) concept.

However, our research also has several limitations. First, even though the admittedly commercial antibody mAb 11E7 and some experimental samples were used to evaluate our two serological methods, the lack of testing with field samples is a notable limitation of this study. Our future work will validate the developed tests using clinical samples collected from CCHFV-endemic regions, particularly in areas in China where sheep and ruminants have been reported to be positive for the virus. Second, the two serological methods to detect the specific antibody against CCHFV GP in serum samples were developed, but the reliability and usefulness of the two assays should be evaluated by comparing the results of widely used traditional ELISA and “gold-standard” neutralization assay using inactivated or infectious CCHFV. Further research should focus on obtaining field samples and conducting extensive validation studies to assess the sensitivity, specificity, and reliability of the rGP-based ELISA and rVSV/CCHFV-based sVNT. Third, choosing the truncated recombinant GP as an antigen may have its limitations, which may not allow the detection of antibodies targeting other GPC epitopes. A comparison of the detection efficiency between truncated recombinant GP and recombinant full-length Gc antigen protein is needed. Finally, as the glycoprotein of different CCHFV strains exhibits genetic variability, with the mucin-like domain in G_N_ in particular being highly divergent and even the highly conserved G_C_ showing antigenic variability, whether the rGP and rVSV/CCHFV in the present study can detect samples of different CCHFV strains is the direction of our future efforts. We are always working to find highly conserved antibody-binding sites and establish surrogate viruses of different strains to develop universal detection methods for IgG and neutralizing antibodies.

## 5 Conclusions

Our bacterially expressed glycoprotein and recombinant virus based on vesicular stomatitis virus have good antigenicity, and the novel rGP-based IgG ELISA and surrogate virus (rVSV/CCHFV)-based neutralization assays can be safe tools for detecting antibodies against CCHFV GP including commercial inhibitors and inactivated animal sera in a BSL-2 laboratory. The use of the broadly cross-reactive and neutralizing antibody clone 11E7 further strengthens the reliability and specificity of the assays. Therefore, the rGP and rVSV/CCHFV are promising alternatives to the CCHFV for research on specific antibody and serological samples.

## Data availability statement

The raw data supporting the conclusions of this article will be made available by the authors, without undue reservation.

## Ethics statement

The studies on the sVNT using the recombinant virus (rVSV/CCHFV) were performed under BSL-2 conditions. All rodent experiments were strictly conducted according to the guidance of the Animal Welfare and Ethics Committee of the Changchun Veterinary Research Institute, Chinese Academy of Agricultural Sciences(approval number: IACUC of AMMS-11-2020 -020). cDNA encoding the open reading frame of the CCHFV IbAr10200 strain GPC (GenBank ID: AF467768) was synthesized by Sangon Biotech (Shanghai) Co., Ltd. (Shanghai, China).

## Author contributions

QW: Formal analysis, Software, Writing – original draft. SW: Methodology, Writing – review & editing. ZS: Methodology, Writing – review & editing. ZL: Methodology, Writing – review & editing. YZ: Conceptualization, Writing – review & editing. NF: Conceptualization, Writing – review & editing. TW: Conceptualization, Writing – review & editing. FY: Conceptualization, Funding acquisition, Project administration, Writing – review & editing. XX: Conceptualization, Supervision, Writing – review & editing.
